# Valuable Contributions

**Published:** 1883-02

**Authors:** 


*Valuable Contributions* :—The Museum of the Dental Department of the University of Maryland has recently been presented with several entire dentures of "Continuous Germ Work" by Dr. John Allen, of New York City. These valuable specimens are gotton up in the beautiful and attractive manner for which Dr. Allens' work has so long been noted, and are perfect specimens of what may be accomplished in a style of dental mechanism which has always commanded the admiration of dental practitioners and patients. Demonstration and work by students in 'Continuous Germ' have been conducted in the Laboratory of the Dental Department of the University of Maryland during the present session, and some excellent specimens of work have been the result.

Dr. Samuel A. White, of Savannah, Georgia, has presented to the same Dental Museum beautiful casts in plaster of a rare form of irregularity, in which the molars alone articulate, the teeth anterior to them standing apart, so that the space between the incisors of both jaws is over one inch in extent. Dr. White has also presented a number of specimens of malformed teeth which are both interesting and unique.

Dr. W. T. Pool, of Columbus, Georgia, has also presented to this Museum with several hundred specimens of abnormally shaped teeth.

So many and valuable are the specimens already collected that the Museum of the Dental Department of the University of Maryland will already compare favorably with those of other institutions, and will soon excell many of them in the nature and value of its specimens.

